# Commentary: Can Ordinary People Detect Deception after All?

**DOI:** 10.3389/fpsyg.2017.01789

**Published:** 2017-10-13

**Authors:** Chris N. H. Street, Miguel A. Vadillo

**Affiliations:** ^1^Department of Psychology, University of Huddersfield, Huddersfield, United Kingdom; ^2^International Research Centre for Investigative Psychology, University of Huddersfield, Huddersfield, United Kingdom; ^3^Departamento de Psicología Básica, Universidad Autónoma de Madrid, Madrid, Spain

**Keywords:** lie detection, unconscious, tipping point, indirect lie detection, unconscious lie detection, unconscious thought theory, deception detection

No one likes to call someone a liar. But the authors of the tipping point account (ten Brinke et al., 2016) claim that it is evolutionary prudent to spot lies that can harm us in order to determine who to trust. As such, they propose the reputational costs of confronting a liar might be overcome by detecting lies unconsciously. When confronted with information that creates a threat response, the unconscious can use the threat response to detect deceptive cues and to unconsciously infer deception, all the while keeping this information out of the conscious mind. The account suggests this is beneficial because conscious awareness of the deception “could impel the perceiver to confront the liar” (p. 580).

The account is controversial insofar as it claims that people can detect deception, in contrast to past work showing otherwise (47% detection rate of lies, and 61% of truths, resulting from bias to judge statements as true: Bond and DePaulo, 2006), and also makes novel claims about an unconscious ability. Although it is welcoming to see new theoretical approaches to lie detection, the account (a) makes claims that do not match the data and conclusions presented in the studies cited to build its case, (b) offers no testable definition of unconscious processes, and (c) contains internal contradictions.

The first issue is with the mismatch between what the studies found and what the tipping point authors are interpreting from them. For instance, ten Brinke et al. (2016) interpret work on nonhuman animals (primates and canines) as showing that nonconscious thinking can detect deception (Wheeler, 2010; Takaoka et al., 2015), and predict that, for example, “Canine behavior will reveal a preference for approaching truth-tellers and avoiding liars” (p. 582). However, the cited works do not explore deception or lie detection. Takaoka et al. (2015) trained dogs to go to a container that concealed food, identified by a person pointing at the correct container. After training, the dogs are shown which container is baited, and then a person points to the wrong container. The dogs correctly choose the baited container. Is the canine drawing on knowledge of deception, or is this evidence that dogs do not use unreliable information when they have more robust information available (i.e., having seen which container is baited)? We, and the original authors, would argue for the latter. Similarly, ten Brinke et al. cite Wheeler (2010) in support of the claim that “[n]onhuman primates can detect deception at higher rates than humans” (p. 582). But this study does not test deception or lie detection, let alone compare human and primate performance.

In the same vein, neuropsychological work is cited (Grèzes et al., 2004; specifically, Grèzes et al., 2006; Lissek et al., 2008) to argue that brain- or body-based physiological responses occur when observing deception. This may seem to suggest that people are unconsciously responding to deception. But in these studies participants were *explicitly* made aware of the possibility of deception and were asked to make lie-truth judgments, sometimes reaching 100% accuracy. It is not clear how one would show that the physiological activity is not indicative of the conscious judgment they were asked to make.

The largest body of evidence supporting unconscious lie detection stems from the indirect method. Participants are not consciously informed about the possibility of deception. Instead, they judge whether the speaker, for instance, appears to be thinking hard. These studies find that judgments of thinking hard (or some other indirect judgment of deception) distinguishes liars and truth-tellers more accurately than an explicit lie-truth judgment. ten Brinke et al. cite work showing that people feel less comfortable and more suspicious (two indirect judgments) when viewing their friends' deceptions compared to viewing their truths, but were at chance accuracy in making an explicit lie-truth judgment (Anderson et al., 2002). It would appear that the rater cannot explicitly distinguish lies from truths, but feels uncomfortable when listening to lies, which might suggest some form of unconscious knowledge. However, Anderson and colleagues demonstrated that their result was a methodological effect attributable to the fact that the scale used to collect explicit ratings was less sensitive than the one used for indirect ratings, an effect which has been found in a meta-analysis (Bond and DePaulo, 2006). In fact, indirect lie detection often performs worse than direct lie detection (Levine and Bond, 2014; Bond et al., 2015), and can be explained by entirely conscious processes (Street and Richardson, 2015; Street and Vadillo, 2016).

To the best of our knowledge, only two of the studies cited by ten Brinke et al. (Reinhard et al., 2013; ten Brinke et al., 2014) contain unambiguous evidence in favor of unconscious lie detection. But the reliability of these two findings has been called into question by failures to replicate the former (Moi and Shanks, 2015) and several oddities in the analysis of the latter (Levine and Bond, 2014; Franz and von Luxburg, 2015).

The second issue with the tipping point account is its falsifiability. The authors offer two and a half pages of predictions, but unfortunately, *none* of them test whether the effect is unconscious. For instance, it is predicted that, “[e]xperiencing social exclusion will enhance accuracy” (p. 583). If this prediction was supported, we cannot know whether it arises from unconscious thinking. The authors do not explain what the unconscious is or how it is possible to test whether the unconscious is involved. There is an active and ongoing debate around whether the unconscious exists (e.g., Newell and Shanks, 2014). Because of the lack of a definition of what the unconscious is, how it could be measured, or how it should work, the tipping point theory's claim to the unconscious is unfalsifiable.

The third issue with the account is that there are a number of inconsistencies. For example, it is predicted that increasing reputational and relationship costs of accusing others of deception should detriment accuracy: “When social norms shift and license people to catch liars, thus attenuating the social costs of declaring someone a liar, accuracy improves” (p. 586). But the account also attempts to harness findings showing that when the costs to the relationship are perceived to be particularly high, accuracy actually *improves*, citing Ein-Dor and Perry (2013). It seems difficult to reconcile these two contradictory positions.

While a threat to the self may engage the unconscious to help detect the lie, an overwhelming threat may lead people to be suspicious and judge whatever they hear to be a lie, even at the expense of accuracy. What is an overly potent threat? The authors cite work showing that police officers are biased to judge “lie” when rating footage of students committing mock theft and vandalism (Meissner and Kassin, 2002). If this is sufficiently threatening to overwhelm any accuracy effects, the level of threat that the unconscious has evolved to detect seems particularly benign. Yet deception gets people to offer up their financial details (Wright et al., 2010) and being person-trafficked (United Nations Office on Drugs and Crime, 2004; Hübschle, 2014). These are potent threats that should create a lie bias, but people seem to believe the persuader.

The tipping point account acknowledges that the threat response only allows higher accuracy “when cues to deception are present and perceptible” (ten Brinke et al., 2016, p. 580). That threat may make people judge statements as lies (a “lie bias”) is consistent with current theories that do not rely on unconscious processing. The effect of threats creating a lie bias is consistent with the context-general information use of ALIED theory (Street, 2015) and with the concept of triggers in truth-default theory (Levine, 2014), neither of which require a claim to the unconscious. The suggestion that the social repercussions of accusing others may cause a truth bias has been made by O'Sullivan et al. (1988) and O'Sullivan (2003) without claiming there is an unconscious element. While the accusatory reluctance position has been suggested in the literature, it has received little direct empirical testing. A useful contribution of the tipping point theory, then, is to make explicit a number of predictions that could test for the presence of accusatory reluctance (see Box 1). But this exploration can take place without reference to an undefined hidden process (Street and Vadillo, 2016).

Box 1 **Developing the tipping point account.**

If the account aims to make an unconscious claim, it would benefit from (a) defining what “unconscious” means and how it is supposed to increase accuracy, (b) outlining predictions that test its unconscious claims, and (c) couching the discussion of the unconscious in the unconscious cognition literature. However, given the lack of support from the cited research, the internal inconsistencies that may in part be remedied by removing the claim to the unconscious, and the lack of a testable definition, the account would likely benefit from making no claims to the unconscious.

Given that the predictions may just as easily be accommodated by conscious processes, why does the account argue for an unconscious process? The authors suggest that “if cues to deception enter into consciousness, they could impel the perceiver to confront the liar.” (p. 580). But, consciousness is not impelled to communicate. People are capable of holding conscious thoughts without making them public. In fact, one might even call this a definition of deception: To be aware that what one is saying does not match with what one believes to be true.

## Author contributions

CS and MV contributed approximately equally to the conception, research, and writing of this commentary.

### Conflict of interest statement

The authors declare that the research was conducted in the absence of any commercial or financial relationships that could be construed as a potential conflict of interest.
